# From colonizer to culprit: genomic and clinical insights into *S. epidermidis* from post-surgical endophthalmitis

**DOI:** 10.1007/s10096-025-05206-5

**Published:** 2025-07-05

**Authors:** Susanna Sagerfors, Leandro Andrés Escobar-Herrera, Thor Bech Johannesen, Marc Stegger, Bo Söderquist

**Affiliations:** 1 Department of Ophthalmology, Faculty of Medicine and Health, Örebro University, Örebro, 701 82 SE Sweden; 2 Department of Sequencing and Bioinformatics, Statens Serum Institut, Copenhagen, Denmark; 3 European Society of Clinical Microbiology and Infectious Diseases (ESCMID) Study Group for Staphylococci and Staphylococcal Diseases (ESGS), Basel, Switzerland; 4 School of Medical Sciences, Faculty of Medicine and Health, Örebro University, Örebro, 701 82 SE Sweden; 5 Antimicrobial Resistance and Infectious Diseases Laboratory, Harry Butler Institute, Murdoch University, Perth, WA Australia; 6 Department of Laboratory Medicine, Clinical Microbiology, Faculty of Medicine and Health, Örebro University, Örebro, 701 82 SE Sweden

**Keywords:** *Staphylococcus epidermidis*, Endophthalmitis, Antibiotic resistance

## Abstract

**Purpose:**

To describe the clinical characteristics of exogenous episodes of endophthalmitis from which *Staphylococcus epidermidis* was isolated by vitreous cultures. We also explored the genomic traits of these *S. epidermidis* isolates and their relatedness to *S. epidermidis* originating from carriers and from prosthetic joint infections in the same geographical region.

**Methods:**

*S. epidermidis* isolated from cases of exogenous endophthalmitis (*n* = 33) were genome sequenced. Clinical features were retrospectively collected from medical records. The isolates were compared with previously sequenced *S. epidermidis* isolates from the nares of healthy individuals (*n* = 151) and from prosthetic joint infections (*n* = 138).

**Results:**

The most common ophthalmological procedure preceding the endophthalmitis was a posterior segment surgery (76%; 25/33), mainly intravitreal injection (70%; 23/33). These patients displayed a significantly shorter time to symptoms compared to those with an anterior segment surgery (median 3 vs. 9 days; *p* < 0.001), and significantly less phenotypic methicillin resistance (8%, *n* = 2/25 vs. 50%, *n* = 4/8; *p* = 0.02). Most isolates of *S. epidermidis* originating from endophthalmitis cases did not belong to known healthcare-associated lineages and did not cluster with isolates from prosthetic joint infections. Rather, they were more similar to isolates from the nares of healthy individuals.

**Conclusions:**

Genomic data suggest that the *S. epidermidis* isolated from the vitreous of Swedish cases of postsurgical endophthalmitis may originate from the commensal flora of the individual, and not from the healthcare facilities. The type of preceding surgical procedure (anterior vs. posterior segment) may influence both symptom delay and the presence or absence of methicillin resistance.

**Supplementary Information:**

The online version contains supplementary material available at 10.1007/s10096-025-05206-5.

## Introduction

Bacterial endophthalmitis is a potentially devastating but rare intraocular infection, characterized by inflammation, visual loss, and pain. Exogenous transfer of bacteria during a surgical procedure or via penetrating trauma is the most common pathway for the bacteria to enter the eye [1, 2]. One of the most frequently isolated bacteria in exogenous endophthalmitis following intraocular surgery is *Staphylococcus epidermidis* [3–5]. This Gram-positive aerobe bacterium colonizes human skin and mucous membranes, including the conjunctiva. *S. epidermidis* is an omnipresent human skin and nares commensal, but also an opportunistic pathogen that thrives in healthcare environments, particularly under conditions of antibiotic pressure and invasive procedures involving artificial implants [6]. It is associated with infections of prosthetic devices such as arthroplasties [7], heart valves [8], and intraocular lenses [9].

The ability of *S. epidermidis* to survive and thrive in nosocomial settings is partly due to its ability to adapt to external environmental factors through mutations and acquisition of mobile genetic elements. Examples of this include the staphylococcal chromosome cassette (SCC*mec*), which mediates antibiotic resistance [10], and insertion sequences (IS) such as IS256, which is linked to both biofilm formation and antibiotic resistance [11]. Biofilm production, a key pathogenic mechanism in *S. epidermidis* foreign-body infections, is facilitated by polysaccharide adhesin encoded by the *icaADBC* operon, which is associated with specific nosocomial clonal lineages [12, 13]. However, *icaADBC*-negative strains have been shown to produce biofilm through proteins such as accumulation-associated protein (Aap) and biofilm-associated protein (Bap/Bhp) [14–16].

Globally, the predominant healthcare-associated lineage is *S. epidermidis* sequence type 2 (ST2). Lee et al.. proposed that the diversification of the ST2 lineage began in the 1950s, with the emergence of a rifampicin-resistant sub-lineage in the 1970s [17]. In Sweden, ST2 strains have been identified as predominant in prosthetic joint infections (PJIs) [18] and in bacteraemia among patients with haematological malignancies [19].

*S. epidermidis* isolated from the aqueous or vitreous of patients with endophthalmitis after cataract surgery have a higher prevalence of antibiotic resistance genes, such as *mec*A, and biofilm-associated genes, such as *ica*A and *aap*, compared to conjunctival isolates of non-infected patients [20]. However, the molecular epidemiology of *S. epidermidis* obtained from patients with endophthalmitis remains largely unexplored.

This study aimed to describe the clinical characteristics of exogenous episodes of endophthalmitis from which *S. epidermidis* was isolated by vitreous cultures. We also explored the genomic traits of these *S. epidermidis* isolates and their relatedness to *S. epidermidis* originating from carriers and PJIs in the same geographical region.

## Materials and methods

### Patients

This retrospective descriptive study was approved by the Swedish Ethical Review Authority (2018/120; 2021 − 01134). Eligible patients who underwent treatment for endophthalmitis at the Department of Ophthalmology, Örebro University Hospital, Sweden, a vitreoretinal referral centre, were identified through searching the database of the Department of Laboratory Medicine at the same hospital. The search criteria were “*S. epidermidis*” and “vitreous” for the period between 2015 and 2020. All patients with suspected endophthalmitis treated at the Department of Ophthalmology undergo vitreous sampling for culture, and are given simultaneous intravitreal (2 mg) and subconjunctival (100 mg) injection of ceftazidime along with intravitreal (1 mg) injection of vancomycin [21, 22] and subconjunctival betamethasone (12 mg).

Patient charts and laboratory reports, including antibiotic susceptibility patterns, were reviewed. Patients were included if they fulfilled the criteria for endophthalmitis adapted from Seal et al. [23]; that is, vitritis in combination with at least one of the following symptoms and/or signs: pain, swelling, loss of vision, hypopyon, chemotic conjunctiva, or oedema of the lids, conjunctiva, or cornea. The exclusion criteria comprised toxic anterior segment syndrome according to Mamalis et al. [24], or endogenous episodes of endophthalmitis.

The episodes of endophthalmitis were subdivided into two groups, depending on whether the preceding surgical procedure was restricted to the anterior segment (cataract surgery, refractive surgery, and corneal suturing or removal) or the posterior segment (intravitreal injection [IVI] or vitreoretinal surgery).

Visual outcome was reported in terms of best corrected visual acuity (BCVA; Snellen decimal), both as the median BCVA and sub-grouped as good (≥ 20/40, i.e. ≥0.5) or poor (< 20/40, i.e. <0.5) according to Chiquet et al. [25].

### Microbiology and antibiotic susceptibility testing

Vitreous taps for culture were achieved by trocar and vitrector under sterile conditions in the operating room. Isolated bacteria were determined to species level by matrix-assisted laser desorption/ionization time-of-flight mass spectrometry (MALDI-TOF MS) (Microflex LT and Biotyper 3.1, Bruker Daltonik, Bremen, Germany). *S. epidermidis* isolates were stored pending further analysis in preservation broth (trypticase soy broth with 0.3% yeast extract and 29% horse serum) at -80 °C.

Antibiotic susceptibility was determined by the disc diffusion method or by gradient test, using version 11.0 of the EUCAST clinical breakpoints. Multidrug resistance (MDR) was defined as resistance to three or more antibiotic classes. Details of culturing and antibiotic susceptibility testing are given in the Online Resource.

### DNA purification and sequencing

Bacterial DNA was extracted using an enzymatic pre-lysis step and purified using the MagNA Pure 96 DNA and Viral NA Small Volume Kit, with the DNA Blood ds SV 2.0 protocol (Roche Diagnostics). For each sample, two white inoculation loops filled with colony biomass were suspended in 550 µL of the prepared lysis buffer: 1x PBS, 20 mM Tris-HCl, 2 mM EDTA, 1.2% (w/w) Triton X-100, and 20 mg/mL lysozyme. Following incubation at 37 °C for 30 min, 50 µL of Proteinase K was added to each sample. Samples were then incubated at 56 °C for 30 min. DNA quantification was performed using Qubit (Invitrogen, Waltham, MA, USA). Library and sequencing were prepared per the manufacturer’s guidelines with the Nextera XT Kit (Illumina, Little Chesterford, UK) on the NextSeq 550 (Illumina) platform using 300-cycle kits. Post sequencing, bifrost (https://github.com/ssi-dk/bifrost) was utilized for quality control including sequencing depth (minimum 25-fold coverage), species validation, and detection of contaminants.

### De Novo assembly and typing

To determine the relatedness of the isolates, the raw sequencing data were aligned with the chromosome of *S. epidermidis* strain ATCC_12228 (NCBI accession number NZ_CP022247) using NASP v1.1.0 [26] with BWA-MEM [27] and GATK [28] to identify single nucleotide polymorphisms (SNPs). Positions with < 90% unambiguous variant call and a depth of < 10-fold coverage in individual isolates were excluded across the collection. Gubbins [29] was utilized with default settings on the SNP alignments from NASP to identify and remove recombinant regions. The alignments were then analysed with IQ-TREE v2.1.1 [30] to determine isolate relatedness and to generate a midpoint-rooted phylogeny. Assemblies were generated using SPAdes v3.9.0 [31]. The prediction of sequence types (ST) was identified using mlst [32].

### Sequence analysis – resistance and virulence

Antibiotic resistance genes on the SPAdes-derived contigs were identified using abriTAMR v1.0.17 [33] with the AMRFinderPlus database (2022-08-09.1). ARIBA v2.14.6 [34] was used to identify virulence genes. The ARIBA database was designed to include sequences based on the following reference genes: *aap* (CP000029.1, positions 2 461 683–2 454 490), *bhp* (CP000029.1, positions 2 442 370–2 449 578), *embp* (AY101364.1), *icaA* (U43366.1, positions 761–1999), *sesI* (CP000029.1, positions 1 693 235–1 693 840), *fdh* (NZ_AKGZ01000029, positions 10 249–13 200), *ACME-arcA* (AB817064, positions 4224–5459), IS*256* (CP018629.1, positions 533 639–534 811), *qacA* (MK040371.1, positions 1–1545), and *qacB* (AY028779.1, positions 2550–4094). Only hits with more than 80% sequence similarity and coverage were retained. The presence of specific point mutations associated with antimicrobial resistance was also tested using ARIBA. These included point mutations in *gyrA* (S84F), *parC* (S80F, S80I, S80Y, D84Y), *parE* (N404S), *rpoB* (S464P, D471E, H481Y, I527M, A534V), 23 S rRNA (G2447T, T2504A, C2534T, G2576T, G2603T), *qacA* (A157G), *walk* (P415L, V500F), and *fusA* (L461K).

### Statistical analysis

Statistical analysis of clinical features was performed with the Mann-Whitney U test for comparisons of age, time to symptoms, and BCVA (Snellen decimal). The χ^2^ test or Fisher’s exact test, as appropriate, was used for comparisons on sex, laterality, season at disease onset, and proportion of isolates with reduced antibiotic susceptibility or with methicillin resistance. These analyses were performed in IBM SPSS v25.

Fisher’s exact test was used for statistical analysis of the genomic data, using the R package ‘stats’.

A *p*-value of < 0.05 was considered statistically significant.

## Results

### Study cohort

In total, 35 vitreous cultures with growth of *S. epidermidis* from 34 patients were identified. One episode of suspected endogenous endophthalmitis and one atypical episode with prolonged inflammation after cataract surgery, in which a vitreous sample was collected approximately 8 months after the initial surgery, were excluded. This resulted in a cohort of 33 episodes from 32 patients with exogenous endophthalmitis, in which vitreous cultures showed growth of *S. epidermidis*.

One patient had two episodes of endophthalmitis, both in the same eye, but separated by 6 months. The *S. epidermidis* strains isolated from each episode displayed different susceptibility patterns, and so these two episodes were considered separate, and both were included in the analysis. Both episodes were preceded by IVI with bevacizumab due to neovascular age-related macular degeneration.

Most of the cases (31/33, 94%) were monomicrobial. Of the two episodes with polymicrobial growth, one was preceded by the removal of sutures after corneal transplant performed using Descemet’s stripping automated endothelial keratoplasty. In addition to *S. epidermidis*, the vitreous culture in this case showed growth of alpha-haemolytic streptococci belonging to the salivarius group. The *Streptococcus* isolate showed full susceptibility to all tested antibiotics, while the *S. epidermidis* isolate displayed resistance to the tested fluoroquinolones (ciprofloxacin, levofloxacin, and moxifloxacin) and to beta-lactam antibiotics. The second episode of polymicrobial endophthalmitis was preceded by an uncomplicated cataract surgery. In this case, the vitreous culture revealed *S. epidermidis and Staphylococcus lugdunensis*, both susceptible to all tested antibiotics.

### Clinical features

Clinical characteristics are presented in Table 1. The cohort had a median age of 75 years (range: 54–91) and consisted of 18 women (one of whom had two separate episodes) and 14 men. The right eye was affected in 18 cases (18/33; 58%). Of the 16 eyes with remaining crystalline lenses at the time of endophthalmitis, the episode of endophthalmitis was preceded by IVI in 15 eyes and by corneal suturing after trauma in the remaining eye. Four (25%) of these 16 eyes underwent cataract extraction within 15 days after the initial sampling and treatment for endophthalmitis.

**Table 1:** Clinical characteristics of the 33 episodes of endophthalmitis where Staphylococcus epidermidis was isolated from the vitreous

	All episodes (*n* = 33)	Anterior segment surgery (*n* = 8)	Posterior segment surgery (*n* = 25)	*p*-value
Age	75 (54–91) [71; 81]	75 (54–89) [66; 79]	75 (62–91) [72; 81]	0.527 ^a^
Time to symptoms ^b^	3 (0–17) [2; 4]	9 (3–17) [4; 13]	3 (0–8) [2; 3]	< 0.001 ^a^
Time to sampling/antibiotics ^b^	4 (2–20) [3; 8]	10 (4–20) [5; 14]	4 (2–9) [3; 6]	0.004 ^a^
Visual acuity closest to causing surgery ^c^	0.5 (0.13–1.0) [0.31; 0.62]	0.6 (0.2–1.0) [0.4; 0.8-]	0.5 (0.13–1.0) [0.3; 0.6]	0.259 ^a^
Visual acuity prior to sampling and antibiotics	0.001 (0–0.2) [0.001; 0.001]	0.001 (0–0.01) [0; 0.01]	0.001 (0–0.2) [0.001; 0.001]	0.836 ^a^
Final visual acuity ^d^	0.25 (0–1.0) [0.13; 0.4]	0.7 (0.03–1.0) [0.15; 1.0]	0.2 (0–1.0) [0.1325; 0.31]	0.073 ^a^
Time when final acuity measured ^d^	91 (6–390) [70; 151]	172.5 (75–390) [94; 313]	81 (6–335) [59; 116]	0.012 ^a^
Sex, male	14 (42%)	6 (75%)	8 (32%)	0.047 ^e^
Laterality, right eye	18 (55%)	4 (50%)	14 (56%)	1.0 ^e^
Season at disease onset				
Winter-Spring (Dec–May)	8 (24%)	6 (75%)	2 (8%)	< 0.001 ^e^
Summer-Autumn (Jun–Nov)	25 (76%)	2 (25%)	23 (92%)	
Status findings at presentation				
Hypopyon	26 (79%)	6 (75%)	20 (80%)	1.0 ^e^
Fibrin	14 (42%)	5 (62.5%)	9 (36.0%)	0.2348 ^e^
Absence of fundus reflex ^f^	4 (12%)	1 (20%)	3 (37.5%)	1.0 ^e^
Absence of fundus visibility/details ^g^	21 (64%)	5 (100%)	16 (84.2%)	1.0 ^e^
Final visual acuity according to Chiquet et al.[25] ^d^				
Poor (< 20/40, i.e. <0.5)	24 (73%)	3 (38%)	21 (91%)	0.006 ^e^
Good (≥ 20/40, i.e. ≥0.5)	7 (21%)	5 (62%)	2(9%)	
Antibiotic susceptibility				
Fully sensitive	18 (55%)	3 (38%)	15 (60%)	0.418 ^e^
Methicillin resistant	6 (18%)	4 (50%)	2 (8%)	0.02 ^e^
Multidrug resistant (≥ 3 classes)	5 (15%)	3 (38%)	2 (8%)	0.078 ^e^

Data are given as median (min–max) [percentile] or as n (%). Visual acuity is reported as best corrected visual acuity (Snellen decimal)

^a^ Mann-Whitney U test. ^b^ Time in days, where day 0 = day of the surgery/injection. ^c^ Missing data for 6 cases (5 posterior, 1 anterior). ^d^ Missing data for 2 cases (both posterior). ^e^ Fisher’s exact test. ^f^ Data missing for 20 cases. ^g^ Data missing for 9 cases

Overall, 25 of the 33 episodes (76%) of endophthalmitis were preceded by a posterior segment surgical procedure: IVI (*n* = 23) or vitrectomy and peeling (*n* = 2). In the remaining 8 patients (24%), the disease episodes were preceded by an anterior segment surgical procedure: cataract surgery (*n* = 5), removal of the suture after corneal transplant (*n* = 1), traumatic corneal perforation (*n* = 1), and add-on intraocular lens after refractive lens exchange for correction of remaining ametropia (*n* = 1).

Information on comorbidities was available for 32 (97%) of the 33 episodes. Underlying diseases were hypertension only or in combination with hyperlipidaemia (*n* = 7), pulmonary disorders (*n* = 5, of which *n* = 4 were associated with smoking), diabetes (*n* = 8), cardiac conditions (*n* = 6) such as coronary disease, arrhythmia, and congestion, immunosuppression (*n* = 2), and various other medical conditions (*n* = 11) such as neurological disorders, psychiatric disorders, endocrine disorders excluding diabetes, and prostatic disorders. Only 4 patients were considered completely healthy; that is, not receiving any pharmacological treatment for an underlying condition.

There was a median delay of 3 days (range: 0–17 days) between the surgical procedure and the onset of symptoms in the total group. When divided by type of procedure (anterior vs. posterior segment), a statistically significant difference (*p* < 0.001) was found, with the median delay being 9 days for an anterior segment surgery and 3 days for a posterior segment procedure. Prior to treatment of the episode of endophthalmitis, two patients presented with BCVA of 0.03 and 0.2 respectively, remaining patients presented with light perception, with or without localization (*n* = 6), detection of hand motion (*n* = 23), and counting fingers (*n* = 2) respectively. The median visual acuity in the total group prior to vitreous sampling and treatment was equivalent to detection of hand motion, which according to Holladay [35] corresponds to 0.001 Snellen decimal.

There was no significant difference in final visual acuity between those who underwent an anterior segment surgery (*n* = 8; median 0.7) and those who underwent posterior segment surgery (*n* = 25; median 0.2) (*p* = 0.073; Table 1). However, when visual outcomes were dichotomized according to Chiquet et al. [25], the proportion of patients who ended up with poor visual acuity was significantly higher among those who underwent a posterior segment surgery than among those who underwent anterior segment surgery (91% vs. 38%, *p* = 0.006). The subgroup of patients who underwent anterior segment surgery had a significantly higher proportion of methicillin*-*resistant *S. epidermidis* (MRSE) than those who underwent posterior surgery (4/8 [50%] vs. 2/25 [8%]; *p* = 0.02). A significantly higher proportion of the episodes with an anterior segment surgery had a disease onset during the winter and spring, in comparison to the episodes triggered by a posterior surgery (6/8 [75%] vs. 2/25 [8%]; *p* ≤ 0.001).

### Phenotypic antibiotic susceptibility pattern

The results from the antibiotic susceptibility tests of the 33 *S. epidermidis* isolates are shown in Table 2. Fusidic acid resistance was the most common finding (*n* = 11; 33%). Six (18%) isolates were methicillin-resistant; that is, resistant to beta-lactam antibiotics. Most isolates (18/33; 55%) were susceptible to all tested antibiotics, while resistance to 1, 2, and ≥ 3 antibiotic classes was detected among 8 (24%), 2 (6%), and 5 (15%) isolates, respectively. Of the 5 isolates displaying MDR, 1 was resistant to 3 classes of antibiotics, 1 to 4 classes, and the remaining 3 to 5 classes.

**Table 2:** Antibiotic susceptibility pattern among 33 strains of Staphylococcus epidermidis isolated from the vitreous of patients with exogenous endophthalmitis during 2015–2020

		Aminoglycosides		Amphenicol	Lincomycin	Beta-lactam	Oxazolidinones	Fluoroquinolones			Glycopeptide	Lipopeptide	Dihydrofolate reductase inhibitor	Antimycobacterials
	Fusidic acid	Gentamicin	Tobramycin	Chloramphenicol	Clindamycin	Isoxazolyl- penicillin	Linezolid	Ciprofloxacin	Levofloxacin	Moxifloxacin	Vancomycin	Daptomycin	Trimethoprim- sulpha	Rifampicin
**Number tested**	33	33	33	33	33	33	4	33	28	26	33	4	15	15
**Susceptible**	22	29	30	33	30	27	4	27	23	21	33	4	13	15
**Resistant**	11	4	3	0	3	6	0	6	5	5	0	0	2	0

### Genomic analysis

The isolates were genetically close, with only one outlier showing a large genetic difference compared to the others. The two isolates from the same patient but from separate episodes displayed clear genetic differences, as they were placed far from each other on the tree and had different STs (5 vs. 278).

**Fig. 1:**
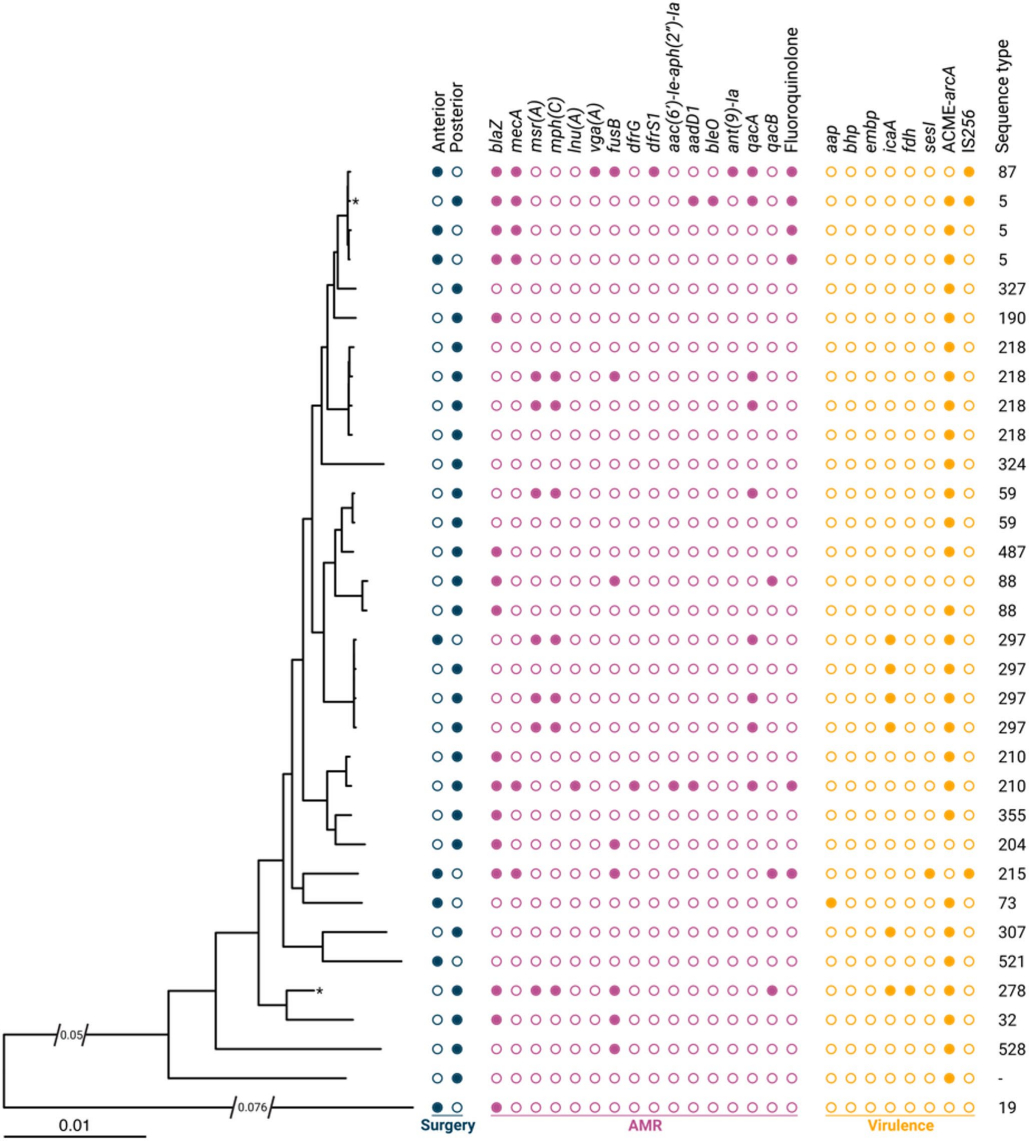
Genomic traits and phylogenetic relatedness of *S. epidermidis* from patients with exogenous endophthalmitis (*n* = 33) based on 66,543 single nucleotide polymorphisms (SNPs). The surgical approach (anterior or posterior) is marked in blue, the presence of antimicrobial resistance (AMR) genes is marked in purple, and the presence of virulence genes, such as genes encoding biofilm, is marked in yellow. The midpoint-rooted phylogeny was inferred using maximum likelihood and was based on a core genome SNP matrix. The scale bar represents substitutions per site. Two edges of the tree had portions cut to add resolution. Where an edge was cut, the length of the cut is marked. Fluoroquinolone resistance was inferred by detection of point mutations (*gyrA*::S84F, *parC*::S80F, and *parC*::S80Y). The asterisks indicate isolates from the same patient but from different episodes separated by 6 months

The most common antimicrobial resistance gene was *blaZ*, identified in 16/33 (48%) of the *S. epidermidis* isolates (Fig. 1). No other resistance determinants were dominant, although several resistance genes were detected, including *qacA* in 9 isolates (27%), *fusB* in 8 isolates (24%), and *msr(A)* and *mph(C)* in 7 isolates (21%). Identical distributions were seen for *mecA* and fluoroquinolone resistance; both were present in 6 (18%) isolates, and neither was present without the other. Moreover, the resistance genes *msr(A)*, *mph(C)*, and *qacA* were generally co-present among the isolated *S. epidermidis*; that is, when one of them was found, the others were likely to also be found. The distribution of STs among the isolates was heterogeneous. The most common STs were ST297, ST218, and ST5, which were represented by 4 (12%), 4 (12%), and 3 (9%) isolates, respectively. Genomic resistance traits were predominantly associated with ST5, ST210, and ST215. The ACME-*arcA* gene was present in 28 of 33 isolates (85%). Other notable virulence genes included *icaA* (6/33; 18%), predominantly detected in the ST297 clade, and IS*256* (3/33; 9%) from ST5, ST87, and ST215.

Six isolates exhibited resistance to fluoroquinolones, with complete concordance observed between their genotypic profiles (Fig. 1) and phenotypic resistance patterns (Table 2).

**Fig. 2:**
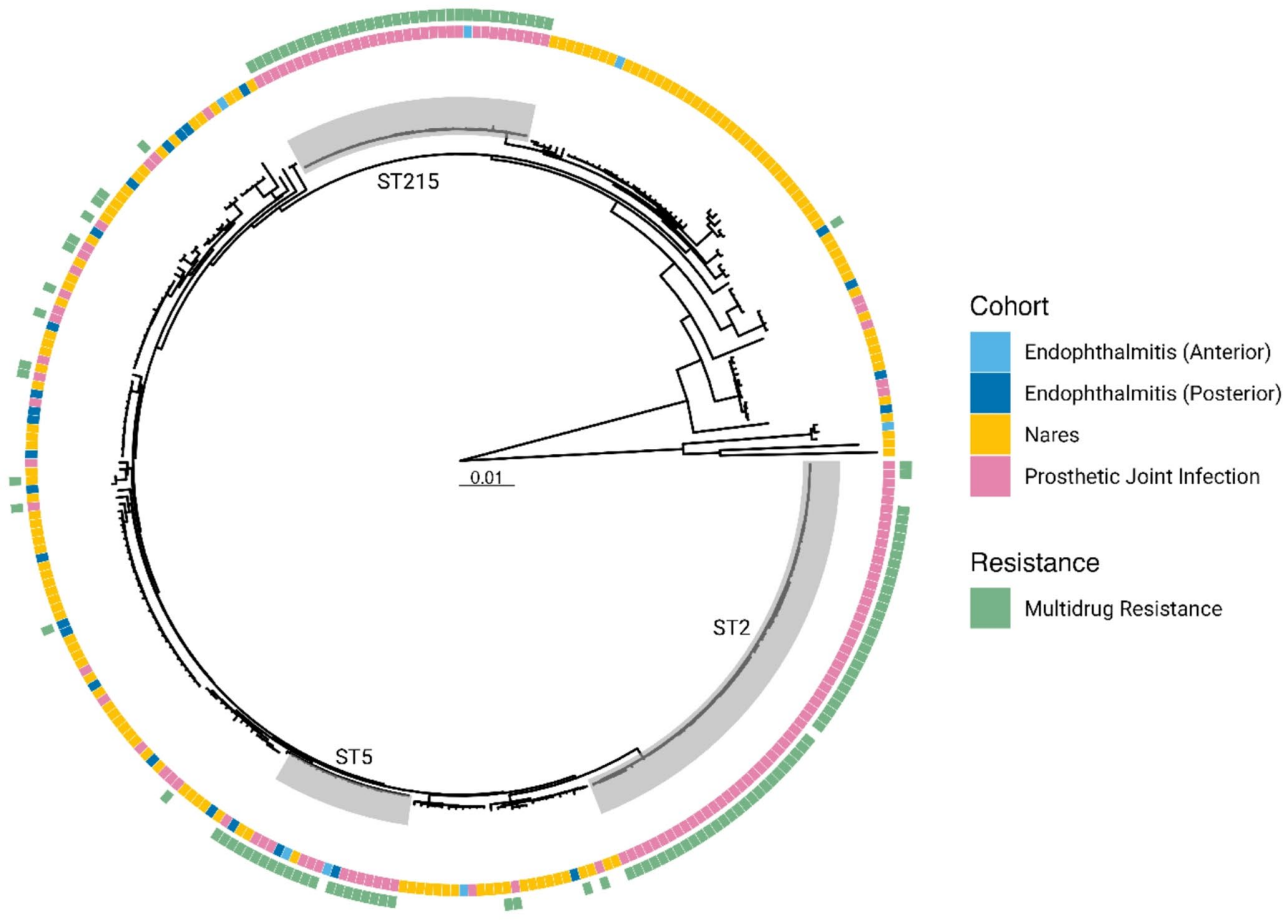
Genomic traits and phylogenetic relatedness of *S. epidermidis* from patients with exogenous endophthalmitis (*n* = 33), prosthetic joint infections (*n* = 138), and nasal swabs from patients scheduled for arthroplasty (*n* = 151). The endophthalmitis samples were divided into two categories depending on the surgical approach (anterior or posterior segment). The sequence types (STs) commonly seen in a hospital environment are highlighted in grey. Multidrug resistance is shown in green and is based on the presence of resistance genes belonging to least 3 antimicrobial categories. The tree was inferred using maximum likelihood, and is based on 63,822 Mb core genome single nucleotides. The scale bar represents substitutions per site

We compared the *S. epidermidis* strains isolated from cases of endophthalmitis with those isolated from PJIs and the nares of healthy individuals, respectively [18] (Fig. 2). All these samples were from the same geographical area of central Sweden. *S. epidermidis* isolates obtained from PJIs were primarily associated with three distinct lineages (ST2, ST5, and ST215), and showed only minimal overlap with samples from the nares and from endophthalmitis cases (Fig. 2). The endophthalmitis isolates did not cluster with the PJI isolates, but instead were dispersed among isolates from the nares. Only a small proportion of endophthalmitis isolates were in the ST5 (3/33; 9%) and ST215 (1/33; 3%) clades, and no ST2 isolates were present. The proportion of genetic MDR among the endophthalmitis isolates (7/33; 21%) was comparable to the proportion in the nares isolates (10/151; 7%) but was significantly lower (*p* = 0.02) than the proportion among the PJI isolates (115/138; 83%). Of the endophthalmitis isolates that displayed genetic MDR, 37.5% (3/8) were isolated from individuals with a preceding anterior segment surgery while 16% (4/25) were isolated from individuals with a preceding posterior segment surgery, but the difference was not significant (*p* = 0.32).

## Discussion

Postoperative endophthalmitis is a rare but devastating complication of intraocular surgery, observed in less than 0.1% of cataract surgeries and in 0.025–0.2% of IVIs [1]. Despite its low incidence, endophthalmitis may affect many individuals due to the large number of procedures performed annually worldwide, including approximately 10 million cataract surgeries [36] and 20 million IVIs [37].

In this study of endophthalmitis with culture-proven *S. epidermidis* from the vitreous, a significantly longer time to presentation was observed among patients who underwent anterior surgery compared to a posterior procedure. This aligns with previous studies of bacterial aetiologies [9, 38]. As reported previously, reasons for this might be that the vitreous provides a more favourable environment for bacterial growth [9, 39] than the aqueous, and that patients who are repeatedly subjected to IVI may be more aware of potential complications and hence more rapidly seek care when experiencing unexpected red-flag symptoms after the procedure [38].

Median BCVA at the last follow-up after the endophthalmitis episode was 0.25 (Snellen decimal) in the total cohort. When BCVA was dichotomized into good (≥ 0.5) versus poor (< 0.5), a significantly higher proportion of the anterior surgery group (*n* = 5; 62%) achieved a good final visual acuity, in comparison to the posterior surgery group (*n* = 2; 9%) (*p* = 0.006; Fisher’s exact test). This confirms the findings from studies on the visual outcome after cataract surgery with isolated CoNS and a mixed microbial aetiology [25, 40, 41].

The proportion of *S. epidermidis* isolates displaying resistance to fluoroquinolones in this study was 15%, which is much lower than the proportions of 37.5–45% reported from Denmark [40] and the US [41]. This may reflect the restrictive antibiotic prescribing policy in Sweden, where topical fluoroquinolones are exclusively prescribed by ophthalmologists.

MRSE accounted for 18% of isolates in this study, which is lower than the rates reported in previous studies of endophthalmitis from the US (53%) and Denmark (30%) [40, 42]. Among cataract surgery cases, 60% (*n* = 3/5) of the isolates displayed methicillin resistance, a proportion similar to the 45% rate of MRSE after cataract surgery-induced endophthalmitis reported in France [25]. The overall lower rate of MRSE in this study may be due to the low proportion of post-cataract-surgery endophthalmitis among the patient population of the present study. Methicillin resistance was significantly more frequent among *S. epidermidis* from anterior surgeries than among isolates from posterior procedures, possibly due to differences in antibiotic prophylaxis. All but one of the cataract patients were given cefotaxime intracamerally (in one case, combined with ampicillin), but only 3/23 IVI patients received any perioperative prophylaxis, and if so, topical chloramphenicol was administered.

In general, the *S. epidermidis* isolates from the vitreous of cases with exogenous endophthalmitis were not carriers of virulence genes, nor were they dominated by common hospital-associated lineages such as ST2. This is in contrast to findings in a French study [20] examining both endophthalmitis following cataract surgery and the conjunctiva of healthy individuals, where the frequency of virulence genes was 32% for *IcaA*, 77% for *aap*, and 18% for IS256, compared to 18%, 3%, and 9%, respectively, in the Swedish cases. The authors of that study also reported a 100% frequency of comorbidity of diabetes among cases with MRSE, compared to 55% among their healthy controls [20], while only 17% (1/6) of the MRSE isolates in the present study originated from a patient with diabetes as a comorbidity. The discrepancy between the reported rates of virulence genes between the French study and the present study may be due to differences between the cohorts, as diabetes was less common in the present study, or due to differences in the distribution of strain types. In the present study, known hospital-associated virulent strains such as ST2 were absent. Strain type was not reported in the French study.

The *S. epidermidis* isolates from the vitreous of cases with endophthalmitis in the present study did not belong to strain types typically associated with the healthcare environment, and hence were not similar to isolates from PJIs in the same geographical region. Instead, they were distributed among strain types found in the nares of healthy individuals from the same region. One possible explanation for this could be the postoperative care practices. The PJI isolates originated from patients who received inpatient care for several days, while patients undergoing the most common triggering ophthalmological surgeries in the present study, IVI and cataract surgery, spent only an hour or two at the healthcare facility and were not admitted. This aligns with the findings of Stefánsdóttir et al.., who reported a significant increase in methicillin and gentamicin resistance among CoNS isolated preoperatively and postoperatively from the groins of patients undergoing joint replacement surgery. They attributed this increased resistance to extended inpatient postoperative care [43].

We found a high frequency (85%) of ACME-*arcA* among the *S. epidermidis* isolates in the present study. This is consistent with previous findings from nostril and nares isolates, which showed a positive rate of 67–76% among *S. epidermidis* [44], but contrasts with a lower prevalence reported in *S. epidermidis* isolates from PJIs [18]. Granslo et al.. concluded in their study on *S. epidermidis* isolated from neonatal blood cultures that the presence of ACME was associated with lower antibiotic resistance and reduced biofilm production when compared to ACME-negative *S. epidermidis* isolates [45]. The high frequency of ACME-*arcA* and the low frequency of virulence and antibiotic resistance genes in the present study are consistent with their findings.

In Sweden, unlike many other countries, chlorhexidine is the recommended preoperative antiseptic agent. Surgical interventions in Swedish eye care are preceded by periocular washing of the skin with 0.5% chlorhexidine alcohol and irrigation of the eye with a 0.05% chlorhexidine solution [46]. This contrasts with the ESCRS guidelines for prevention and treatment of endophthalmitis following cataract surgery, which recommend the use of 5–10% iodine [47]. In the present study, 36% (*n* = 12/33) of the *S. epidermidis* isolates carried either *qacA* (*n* = 9) or *qacB* (*n* = 3), which is much lower than the 71–80% rates reported from PJIs and blood stream infections, respectively [18, 48]. This is in line with the highly different population structure, lacking the usual MDR nosocomial strains of *S. epidermidis*. These findings also support an earlier Swedish study from 2014, which found no evidence that preoperative use of chlorhexidine selected isolates of *S. epidermidis* with decreased susceptibility to chlorhexidine [49].

The *S. epidermidis* originating from episodes of endophthalmitis in this study were genetically diverse and displayed a low frequency of strain types associated with healthcare facilities. Instead, they clustered with isolates from the nares of healthy individuals. This suggests that healthcare facilities are not the primary source of these infections. Rather, the similarities between the *S. epidermidis* isolates from endophthalmitis cases and those from the nares of healthy individuals [18] indicate that the patient’s own commensal flora may be the main source of *S. epidermidis* in cases of endophthalmitis.

In the present study we aimed to describe all cases of exogenous endophthalmitis with *S. epidermidis* isolated from the vitreous, regardless of the preceding/triggering surgical procedure. The cohort was heterogeneous, and so we created subgroups according to surgical procedure: anterior segment procedures (i.e. entering the anterior chamber and possibly introducing bacteria to it) and posterior segment procedures (i.e. entering the vitreous and possibly introducing bacteria to it). The cases were unevenly distributed, with only 8 cases in the anterior segment surgery group and 25 cases in the posterior segment group. As this was a retrospective descriptive study, no power calculation was performed; instead, all cases meeting the inclusion criteria were included. When interpreting the results of the present study, the size of the cohort and the uneven distribution of the cases between the surgical groups needs to be carefully taken into account.

This study had some limitations, primarily due to its retrospective design. As mentioned above, the cohort was heterogeneous and was distributed unevenly between the two subgroups (anterior versus posterior segment surgery). Since the Department of Ophthalmology at Örebro University Hospital is a tertiary vitreous-retinal referral centre, the cases recruited were not limited to Örebro but included patients from across central Sweden. While this may have introduced some regional variability, it also enhances the study’s generalizability. A key strength of the study is the consistency in sampling and culturing, which were performed exclusively at two centralized facilities at Örebro University Hospital.

## Conclusions

Episodes of exogenous endophthalmitis with *S. epidermidis* isolated from the vitreous were predominantly monomicrobial. The type of preceding surgical procedure (anterior vs. posterior segment) appeared to influence symptom onset, visual outcome, and the presence or absence of methicillin resistance. Genomic analyses suggest that the *S. epidermidis* isolates in these cases of exogenous, primarily postsurgical, endophthalmitis likely originated from the patient’s own commensal flora rather than the healthcare facility.

## Electronic supplementary material

Below is the link to the electronic supplementary material.

Supplementary Material 1

## Data Availability

No datasets were generated or analysed during the current study.
