# Supplementary material for: From colonizer to culprit: genomic and clinical insights into S. epidermidis from post-surgical endophthalmitis
Source: Eur J Clin Microbiol Infect Dis. 2025 Jul 5;44(10):2409–20. doi: 10.1007/s10096-025-05206-5 (PMC12484355; doi:10.1007/s10096-025-05206-5)
Supplement: Supplementary file 1 — Supplementary Material 1 [file 10096_2025_5206_MOESM1_ESM.docx]

Supplementary material

**Vitreous culture and susceptibility testing**

One droplet of each of the vitreous fluids was inoculated on GC agar plates (GC Medium Base, Becton Dickinson, Sparks, MD, USA, supplemented with chocolatized defibrinated horse blood) incubated in air with 5% CO_2_ at 36°C for 2 days, on blood agar plates (3.9% Columbia Blood Agar Base, Oxoid, Basingstoke, Hampshire, UK, supplemented with 6% defibrinated horse blood) incubated in air at 36°C for 2 days, on fastidious anaerobe agar plates (4.6% LAB 90 Fastidious Agar, Lab M, Heywood, Bury, UK, supplemented with 5% horse blood) incubated under anaerobic conditions at 36°C for 5 days, on Sabouraud agar plates (1.3% Agar No 2, Lab M; 4% D-Glucose, VWR, Leuven, Belgium; 1% Peptone, Becton Dickinson) incubated in air at 30°C for 7 days, and on CHROMagar Candida plates (CHROMagar, Paris, France) incubated at 36°C for 3 days, respectively. Furthermore, the collected vitreous material was inoculated in BACTEC PED and BACTEC PLUS ANAEROBIC blood culture bottles (Becton Dickinson, Franklin Lakes, NJ, USA) and incubated at 36°C for 14 days.

Antibiotic susceptibility was determined by disc diffusion according to European Committee on Antimicrobial Susceptibility Testing (EUCAST) guidelines (http://www.eucast.org/) clinical breakpoints v 11.0).

The following antibiotics were tested as routine: cefoxitin (30 mg) (as screening for methicillin resistance), chloramphenicol (30 mg), clindamycin (2 mg), erythromycin (15 mg), fusidic acid (10 mg), gentamicin (10 mg), norfloxacin (10 mg), and tobramycin (10 mg) (all discs from Oxoid, Basingstoke, Hampshire, England). Upon request, rifampicin (5 mg), trimethoprim–sulfamethoxazole (25 mg), and/or linezolid (10 mg) were additionally tested. Vancomycin and optionally ciprofloxacin, levofloxacin, moxifloxacin, and daptomycin MIC values were determined using Etest (bioMérieux, Marcy l’Etoile, France) according to the manufacturer’s instructions.
